# An Unexpected Transformation: Malignant Spindle Cell Carcinoma Developed From Primary Basal Cell Carcinoma

**DOI:** 10.7759/cureus.26632

**Published:** 2022-07-07

**Authors:** Erinie Mekheal, Sindhusha Veeraballi, Brooke E Kania, Leena Bondili, Michael Maroules

**Affiliations:** 1 Internal Medicine, St. Joseph’s University Medical Center, Paterson, USA; 2 Internal Medicine, Saint Michael's Medical Center, Newark, USA; 3 Hematology Oncology, St. Joseph’s University Medical Center, Paterson, USA; 4 Hematology Oncology/Internal Medicine, St. Joseph’s University Medical Center, Paterson, USA

**Keywords:** skin malignancy, radiation therapy, basal cell carcinoma, tumor transformation, spindle cell carcinoma

## Abstract

Spindle cell carcinoma (SpCC)/sarcomatoid carcinoma is a biphasic tumor with molecular and histopathological properties of both epithelial and mesenchymal tumors. SpCC usually occurs either in sun-exposed areas like the head, neck, upper extremities, and chest or in the areas of skin with prior radiation exposure or in immuno-suppressed individuals. Cutaneous SpCC is a very rare disease, with only a handful of reported cases so far. SpCC differs from conventional squamous cell carcinoma (SCC) with dermal infiltration of atypical keratinocytes as single cells with hyperchromatic eosinophilic cytoplasm and elongated, pleomorphic nuclei with multiple nucleoli, in contrast to cohesive nests or islands in SCC. The objective of this study is to complete a review of the current literature and present a rare manifestation of malignant SpCC which developed from a localized basal cell carcinoma following excision and radiation therapy (RT) in a 79-year-old female. We plan to elucidate the importance of a timely and accurate diagnosis of this disease in order to maximize treatment options and improve survival outcomes.

## Introduction

Spindle cell carcinoma (SpCC) is an epithelial and mesenchymal tumor that is a variant of squamous cell carcinoma (SCC) that can manifest in sun-exposed areas and can also manifest in areas of prior radiation [1,2]. Cutaneous SpCC is a rare disease, with only a handful of cases reported so far [2]. Our case outlines the rare occurrence of SpCC which developed 40 years after the diagnosis of primary basal cell carcinoma (BCC).

## Case presentation

A 79-year-old female with a history of BCC of the scalp diagnosed 40 years ago in Ukraine, status post excision and radiation therapy with multiple recurrences treated with imiquimod and vismodegib, presented to the office for a new consult after moving to the United States. Due to poor toleration of vismodegib, the patient had been on single-agent imiquimod for the past two years. She denied weight or appetite loss; however, endorsed pruritus at her scalp wound. The patient had xerosis with erythema and hyperpigmentation and thickened skin behind her left ear, hyperkeratotic papules and plaques with dry crusting on her right parietal area, and telangiectasias consistent with previous radiation therapy (RT) treatment.

**Figure 1:**
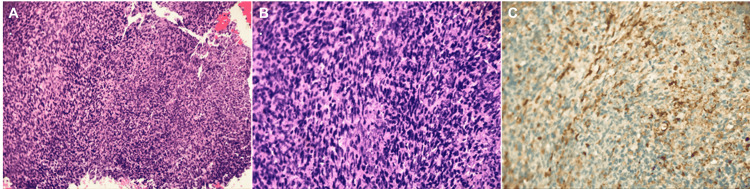
Skin biopsy of the left occipital area with immunostaining A. low power, B. higher power, C. positive for pancytokeratin Skin biopsy images demonstrate spindle cells with extensive necrosis favoring spindle cell carcinoma over squamous cell carcinoma.

The patient received cemiplimab which was held after a few cycles due to hypothyroidism. Cemiplimab was restarted after stabilizing the patient with levothyroxine. She continued to endorse a pruritic scalp lesion notable for a small 1 cm lesion in the left occipital area with another 2 cm lesion in the left parietal area appearing slightly necrotic. The left parietal lesion was biopsied, demonstrating basal cell carcinoma and a left occipital lesion demonstrated malignant spindle cell carcinoma with necrotic changes (Figure 1, Table 1). CT neck, head, chest, abdomen, and pelvis were negative for metastatic disease. Due to the rare and aggressive nature of spindle cell transformation, the patient was referred to the radiation oncologist for consideration of RT.

**Table 1:** Immunohistochemistry analysis

Antibody/Tests	Clone	Results
Pan Cytokeratin	Cytokeratin Pan Type/Cytokeratin 3 Antibody, Anti-Pan Cytokeratin Antibody	Positive
Tumor protein 63	Anti-phosphoserine Antibody	Positive
Cyclin Dependent 34	Anti-Cyclin Dependent 34 Antibody	Negative

**Figure 2:**
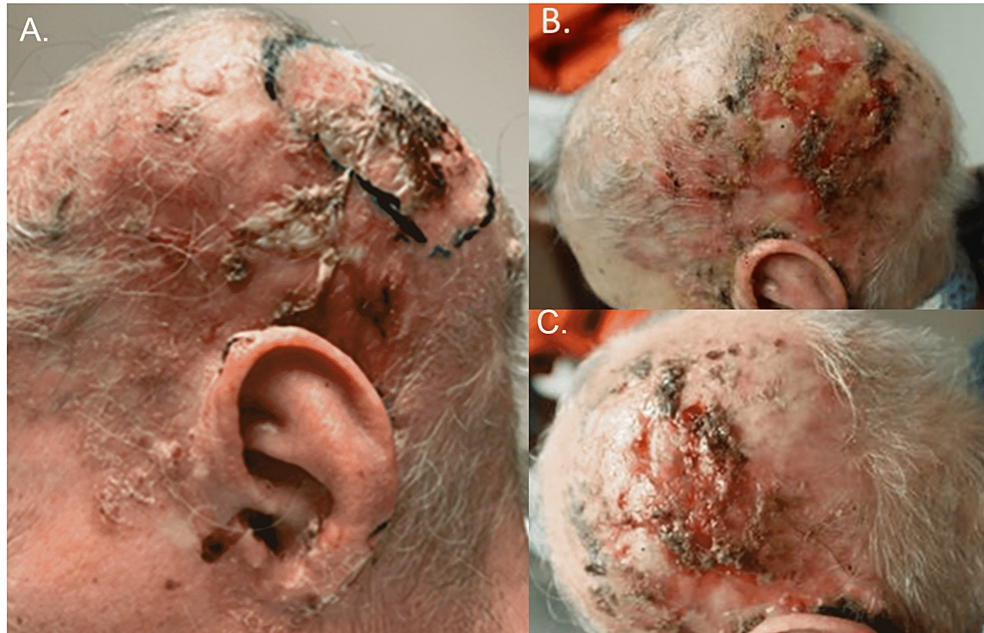
Images of the scalp prior to and following therapy (both systemic and radiotherapy) A. Left posterior scalp image following biopsy yet before the treatment, B. Left posterior scalp image following treatment with chemotherapy and radiotherapy, C. Posterior scalp image following treatment with chemotherapy and radiotherapy

The patient tolerated RT well (Figure 2). She developed dry desquamation during her radiation (attributed to irritation from prior RT), and she was given silver sulfadiazine for this. The patient had continued dry desquamation on her left scalp; and much less necrotic tissue and scabbing, with considerable regression of her right temple and left occipital lesions.

## Discussion

SpCC differs from conventional SCC with dermal infiltration of atypical keratinocytes as single cells with hyperchromatic eosinophilic cytoplasm and elongated, pleomorphic nuclei with multiple nucleoli, in contrast to cohesive nests or islands in SCC [2]. Immunohistochemical studies are essential in the diagnostic discrimination of SpCC from other spindle cell neoplasms. Immunohistochemical studies of SpCC stain positive for a mesenchymal marker, vimentin, and one or more cytokeratins like 34βE12, AE1/3, cam 5.2, or low molecular weight keratin [3,4]. Atypical fibroxanthoma (AFX) differs from SpCC lacking keratin and S-100, and positive CD68. Whereas, spindle cell melanoma can be differentiated with the absence of keratin and the presence of cytoplasmic melanin and S-100. SMA and vimentin are positive, and keratin and S-100 are negative in leiomyosarcoma [4]. In a study analyzing 12 patients with spindle cell squamous cell carcinomas, immunohistochemical studies were positive for 34 beta E12 in 100% cases of SpCC, p63 was positive in 80%, AE1/E3 in 67%, and low molecular weight keratin in 58%, S-100, Desmin, and CD-68 were negative in 100% cases, concluding that the 34 beta E12 was most sensitive in distinguishing SpCC from other histological mimickers [4]. In our patient's case, histologic staining was notable for pan cytokeratin and tumor protein 63, with negativity for cyclin dependent (CD-34), consistent with spindle cell transformation.

Several hypotheses like collision theory, combination theory, composition theory, and conversion theory have been proposed for the pathogenesis of these chimeric biphasic variants of SCC including SpCC, basosquamous cell carcinoma, and carcinosarcoma [5]. In terms of collision theory, it incorporates the idea of two separate tumors that collide, especially when considering an older generation of patients who have had sun-damaged skin and the commonality of both basal cell and squamous cell carcinoma [6]. Alternatively, combination theory demonstrates the sarcomatous and epithelial aspects both derived from a common progenitor cell that underwent differentiation divergently [6]. Additionally, composition theory supports the idea that the stromal component is a pseudosarcomatous response to the malignant epithelial component [6]. Lastly, conversion theory suggests the sarcomatous aspect is a metaplastic transformation of the carcinomatous aspect [6]. This theory is the most widely accepted given genetic monoclonality evidence and carcinosarcoma studies investigating female genitalia [6]. One supporting study investigating immunosuppressed mice demonstrated cutaneous basal cell carcinoma that developed into an anaplastic sarcomatoid tumor, and during various stages of the tumor development, the tumors manifested both sarcomatous and epithelial aspects [7]. In our case and given evidence supporting this theory, conversion theory was considered the likely etiology for this transformation, given metaplastic transformation from a less aggressive to a more aggressive carcinoma. Due to the rarity of the disease, there is no standard treatment protocol available. Additional studies are warranted to identify treatment options to improve clinical outcomes.

## Conclusions

SpCC typically occurs in sun-exposed areas, with the main risk factors including photodamage and ionizing radiation, as in our case. microscopically, SpCC has spindle cells and carcinoma components, with an abrupt or gradual transition between both components. Given the presence of extensive ulceration and necrosis, the spindle cell is usually the dominant component of the tumor, and therefore, the immunohistochemical studies are used to definitively diagnose SpCC as well as differentiate it from other spindle cell neoplasms. Given the rarity of these malignancies, more studies are warranted to identify the treatment option and their clinical outcomes.
